# Stereotaxic surgery for implantation of guide cannulas for microinjection into the dorsomedial hypothalamus in young rats

**DOI:** 10.1016/j.mex.2019.07.005

**Published:** 2019-07-08

**Authors:** Emily I. Poole, Jacob J. McGavin, Nicholas L. Cochkanoff, Karen M. Crosby

**Affiliations:** Biology Department, Mount Allison University, 63B York Street, Sackville, New Brunswick, E4L 1G7, Canada

**Keywords:** Stereotaxic implantation of cannulas in the hypothalamus in young rats, Stereotaxic surgery, Dorsomedial hypothalamic nucleus, Young rats

## Abstract

Stereotaxic surgery to implant guide cannulas into the rodent brain is a frequently used technique to deliver drugs to targeted brain regions in awake, freely moving animals. There are limited reports, however, of central injections in young animals, and no information on cannula implantation for drug administration into the dorsomedial hypothalamus (DMH) in young rats. Our protocol describes a simple and successful method for implanting guide cannulas in the brains of young, male Sprague-Dawley rats and outlines newly developed stereotaxic coordinates to accurately target the dorsomedial hypothalamus.

• Stereotaxic surgical procedure for guide cannula implantation in the DMH in young rats.
• Development of stereotaxic coordinates of the DMH in young rats.
• Microinjection of drugs into the young rat brain.

## Graphical abstract

**Figure:**
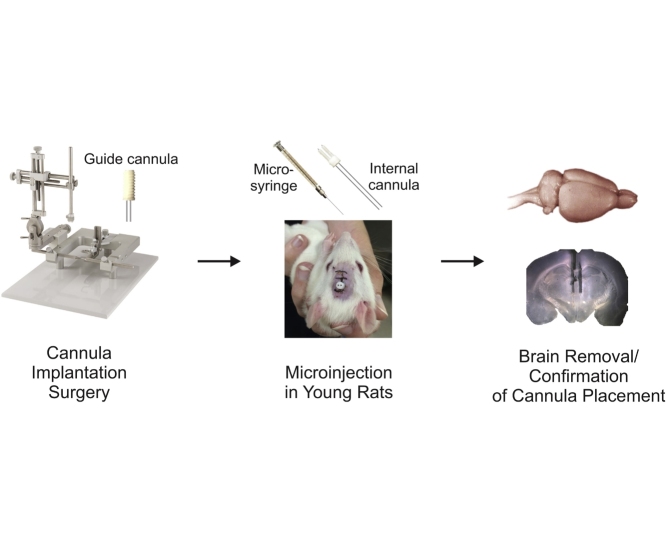

**Specifications Table**

**Table:** 

Subject Area:	Select one of the following subject areas: • Agricultural and Biological Sciences • Biochemistry, Genetics and Molecular Biology • Chemical Engineering • Chemistry • Computer Science • Earth and Planetary Sciences • Energy • Engineering • Environmental Science • Immunology and Microbiology • Materials Science • Mathematics • Medicine and Dentistry • Neuroscience • Pharmacology, Toxicology and Pharmaceutical Science • Physics and Astronomy • Psychology • Social Sciences • Veterinary Science and Veterinary Medicine
More specific subject area:	Stereotaxic surgery
Method name:	Stereotaxic implantation of cannulas in the hypothalamus in young rats
Name and reference of original method:	N/A
Resource availability:	N/A

## Method details

The effects of drugs in specific brain regions can be assessed in awake rodents by surgically implanting guide cannulas that can be later used to microinject drugs into the brain. Although this technique has been used extensively in adult rodents, there are fewer reports in young animals that are more susceptible to surgery-induced mortality and for which detailed stereotaxic coordinates are lacking. Young rats are also extensively used in the neuroscience field for elecrophysiological and other studies that may require stereotaxic injections; thus this technique may be applicable for a wide range of scientists. Here we provide newly developed stereotaxic coordinates for cannula implantation into the dorsomedial hypothalamus (DMH), an important appetite-regulatory center, in young, male Sprague-Dawley rats. We also outline the surgical procedure that yields a less than 2% mortality rate and an 81% success rate of proper implantation in the DMH [1].

## Required equipment

- 1 mm screws
- 2 μL Hamilton microsyringes (×2)
- 24-well plates
- 40 cm connector tubing
- Anesthetic machine with induction chamber and nose cone
- Baxedin Pre-Op (0.5%)
- Clear frozen section compound
- Clippers
- Cryostat
- Dental cement (fast curing custom tray acrylic resin powder and liquid)
- Dental drill with a foot petal; 1 mm and 2.38 mm drill bits
- Dummy cannulas (33 gauge; to fit guide cannulas)
- Electric heating pad
- Ethanol (100%)
- Gauze (sterile)
- Guide cannulas (26 gauge, 1 mm cannula to cannula distance, 8 mm extension below pedestal)
- Guide cannula holder (made on site to fit guide cannulas)
- Hot bead sterilizer
- Internal cannulas (33-gauge, 10 mm extension below pedestal)
- Lamp
- Lubricant (for rectal thermometer)
- Needles (26 gauge)
- Optixcare tear gel
- Oxygen supply for the anesthetic machine
- Paraformaldehyde
- Phosphate buffered saline
- Q-tips
- Rat brain matrix
- Razor blades
- Rodent stereotaxic frame
- Scavenger system and fume hood
- Sodium azide
- Stereo microscope
- Sterile air laminar flow cabinet
- Surgical tools
  • 35 mm serrefines (×4)
  • Forceps
  • Needle drivers
  • Scalpel
  • Scissors
  • Screwdriver for 1 mm screws
- Syringes
- Thermometer (rectal)
- Ultra fine point permanent marker
- Underpads
- Xylocaine (2%)

## Procedure

### Stereotaxic surgery

**Fig. 1:**
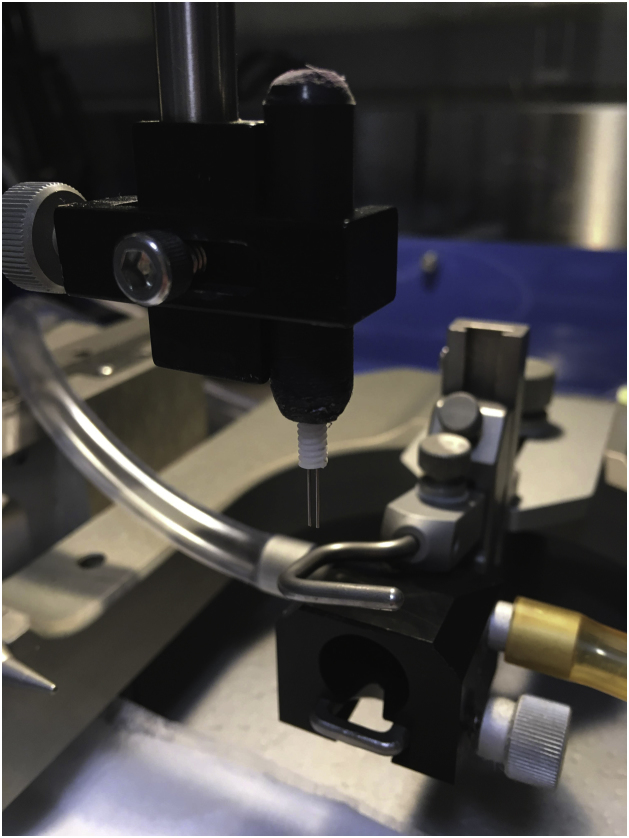
Guide cannula secured to arm of the stereotaxic frame. We made our own holder using a small piece of plastic that we designed to screw on to the top of the guide cannula. Prior to surgery, the guide cannula (with dummy cannula inserted) is screwed into the holder and the holder is mounted onto the arm of the stereotaxic frame with a screw.
- Prepare sterile physiological saline (2 ml; 0.9%) and warm to ˜37 °C
- Prepare syringes containing 0.1 ml of 0.1 mg/kg buprenorphine

Stereotaxic surgical procedures are carried out in young (postnatal day 28–30), male rats weighing approximately 100–120 g between 8 h00–12 h00. In preparation for surgery, the following steps are taken (beginning approximately 40 min prior to planned anesthesia time):

- Turn on heating pad and lamp
- Sterilize all surgical instruments and equipment using Baxedin Pre-Op (0.5%) and ethanol (100%) and place surgical instruments in a hot bead sterilizer for 15 s
- Set up the stereotaxic frame to ensure rat is positioned in the center of the frame
- Add isoflurane to the anesthetic machine
- Insert dummy cannula into guide cannula and screw the guide cannula into a holder and secure this to the arm of the stereotaxic frame (Fig. 1; we made a black plastic holder that precisely holds the guide cannula)

Following preparation, turn on the isoflurane machine and ensure that the flow rate of oxygen is ˜0.8 L/min. Fill the induction chamber with 0.5% isoflurane in oxygen for two minutes. Place the animal in the induction chamber and then increase the percentage of isoflurane in 0.5% increments every 20 s until it reaches 3.5%. Keep the animal in the chamber at 3.5% for an additional 2–3 min until a deep level of anesthesia is achieved. Transfer the animal to a nose cone attached to the bite plate of the stereotaxic frame and carefully move the tongue to the side while ensuring the top incisors are over the bite plate. The animal should be placed on an underpad on a towel on top of an electric heating pad. Confirm a lack of sensation with a toe pinch and then center and secure the animal with the ear bars of the stereotaxic frame. Once the ear bars are securely in place, decrease the percentage of isoflurane and maintain an isoflurane concentration of 2.5–3.0% for the remainder of the procedure. Insert a lubricated rectal thermometer and carefully monitor body temperature throughout the procedure. Keep the heating pad warm enough so that the animal’s core body temperature does not drop below 37 °C. If the temperature rises above 37.5 °C, turn off the heating pad. Using clippers, clear a section of the head from the neck to just posterior to the eyes and apply an antiseptic (Baxedin Pre-Op; 0.5%) and a topical anesthetic (xylocaine; 2%) to the shaved region to sterilize and anesthetize the region. Apply tear gel (Optixcare) to the eyes to prevent them from drying out. Administer 2 ml of warmed sterile physiological saline (0.9%; we dropped our mortality rate to almost 0% when we started administering 2 ml of saline instead of 1 ml) and 0.1 ml of buprenorphine (0.1 mg/kg) in separate subcutaneous injections in the subscapular area.

Use a scalpel to make a small incision (2 cm) medial to the eyes and beginning at the posterior region of the eyes. Clip open the incision to expose the skull using 4 × 35 mm serrefines. Clear away the blood and other connective tissue from the surface of the skull using Q-tips until the skull is dry and bregma, a landmark on the skull where the coronal and sagittal sutures converge, is clearly visible. Position the bilateral guide cannula above the most posterior part of bregma and then move the cannula to the following coordinates that we have confirmed correspond with the DMH in young rats: 2.0 mm posterior and ±0.5 mm lateral (lateral coordinates are naturally achieved if the bilateral cannula with 1 mm spacing is properly centered along the midline). Note: we have also successfully implanted cannulas in the DMH with the cannula positioned 2.25 mm posterior to bregma; with this coordinate, the cannulas are typically positioned from mid to posterior DMH. When the cannula has been positioned in place using the anterior-posterior and medial-lateral coordinates, draw a box on the skull immediately around the cannula tips using an ultra fine point permanent marker to indicate the target insertion site. Record the exact position of the stereotaxic arm holding the guide cannula and then move it aside to allow room for drilling. Using an electric drill with a 1 mm bit, drill a small hole in the skull anterolateral to the target insertion site and insert a 1 mm screw (1 mm diameter) into the skull to serve as an anchor for dental cement. Next, drill a hole at the target insertion site (indicated by the box) using a 2.38 mm drill bit. Return the guide cannula to the previously recorded position and from the surface of the skull, lower the cannula 6.0 mm into the brain. Note: we have also successfully reached the DMH using a coordinate of 6.75 mm ventral to the skull surface. This coordinate will position the guide cannula approximately 2 mm above the DMH. This prevents the guide cannula from potentially damaging the target area, and allows for the internal cannula (which extends 2 mm below the guide cannula) to reach the top of the DMH during drug injection. The final stereotaxic coordinates for the implanted guide cannula should be (relative to bregma): 2.0 mm posterior, ±0.5 mm lateral, and 6.0 mm ventral (the latter is from the surface of the skull). For comparison, the coordinates often used in adult animals are 3.1–3.3 mm posterior, ± 0.5 mm lateral, and 6.8–9.0 mm ventral [[2], [3], [4]].

**Fig. 2:**
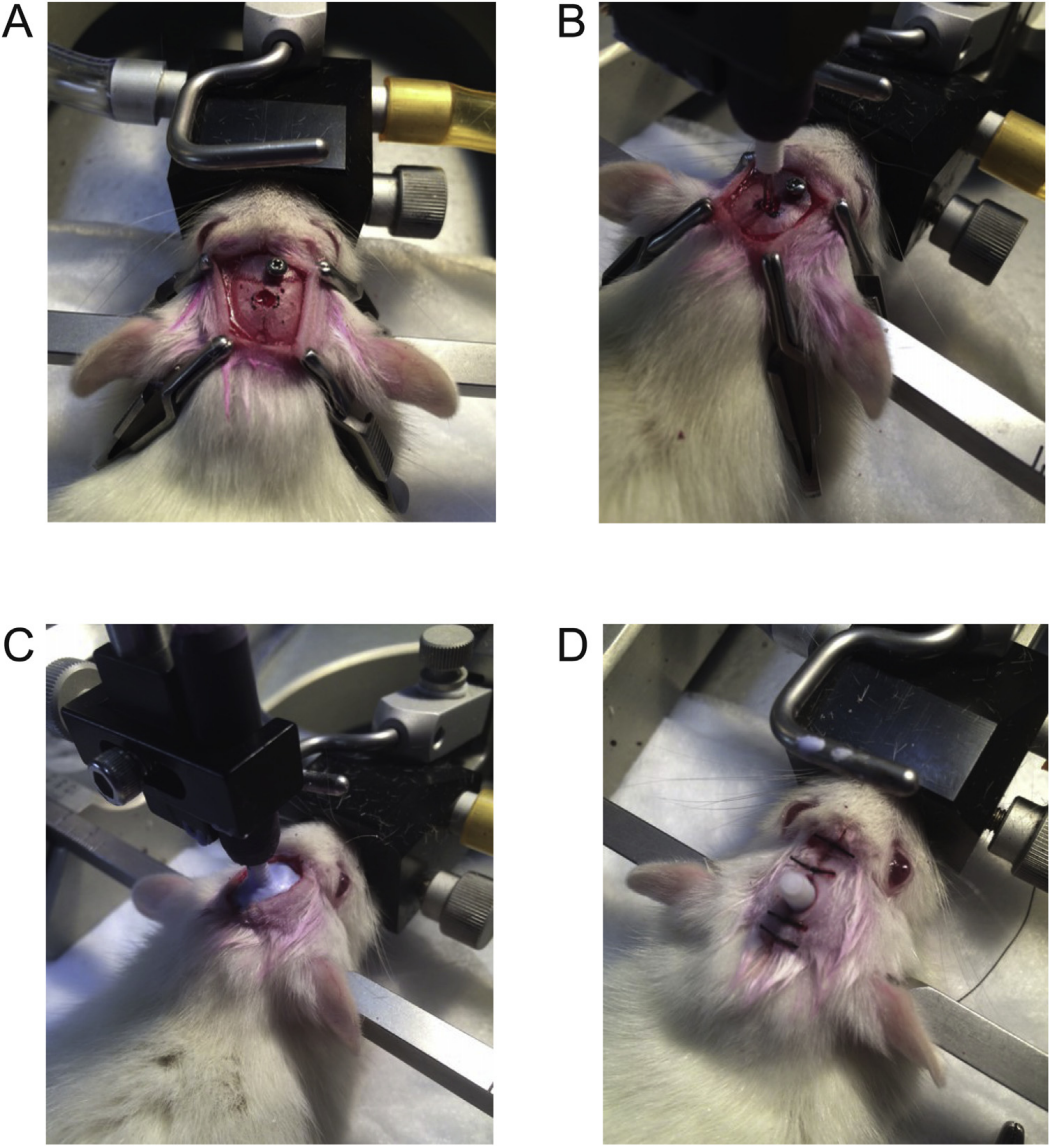
Steps of cannula implantation. A) 1 mm screw inserted anterolateral to the guide cannula insertion site (indicated by the drilled hole). B) Cannula in position to be lowered to the ventral coordinates. C) Cannula inserted in the brain and secured to skull with dental cement. D) Incision site sutured.

Ensure the skull is completely dry and then apply dental cement with a liquid consistency completely around the screw and cannula to secure both in place. Allow the cement to completely harden, and then remove the stereotaxic arm, leaving just the guide cannula in place. Use a 4-0 silk suture kit with a 19 mm 3/8c reverse cutting needle to stitch up the incision site with a total of four stitches (two anterior and two posterior to the cannula; see Fig. 2 for images of the procedure). Apply xylocaine (2%) on and around the incision and reapply the tear gel before removing the animal from the nose cone. The entire surgical procedure should ideally be less than 45 min, from the moment the animal enters the induction chamber to when it is removed from the nose cone. Place the animal back in its cage (with the cage placed on a heating pad) with some moistened rat chow in the bottom of the cage and carefully monitor the animal until it is awake. To control for post-surgical pain, administer buprenorphine (0.5 mg/kg) subcutaneously 6 and 12 h post-surgery, and then every 6–10 h after that until the animal has recovered (we administer at least four doses of buprenorphine post-surgery).

### Post-surgery

Allow animals to recover for one week following the surgical procedure to ensure that data are not affected by post-operative pain or buprenorphine and to ensure sufficient time to handle animals to limit handling-induced stress. After the first two days of this recovery time, handle the animals frequently and simulate the injection procedure by removing the dummy cannula and attaching connector tubing to the guide cannula. Attaching connector tubing to the guide cannula should be done without insertion of internal cannula. Animals should become comfortable in their home cages with the connector tubing attached. Stress is minimized and intra-DMH injections are most successful when animals have been handled for a minimum of 15 min on each of four consecutive days leading up to the injection. One week following the surgery, microinjections into the DMH can be performed. Connect internal cannulas to 40 cm long connector tubing that are pre-filled with deionized water. Use two 2 μL Hamilton microsyringes to draw up ˜0.7 μL of air (this will create an air bubble, movement of which can be measured with a ruler and used to verify successful injection). Fill the remaining ˜1.3 μL with the desired drug in preparation to inject 0.5 μL of drug per side to the DMH. This volume has been used for microinjections into the DMH and other hypothalamic nuclei [2,[5], [6], [7]]. Remove the dummy cannula from the guide cannula in the animal and insert the internal cannula (that will extend 2 mm below the guide cannula to reach the DMH) attached to the connector tubing and syringes. Return the animal to its cage and place the connector tubing and two syringes on a platform beside the cage. Slowly deliver the drug (0.5 μL/side) over a period of 2 min and leave the internal cannula in place for an additional 2 min to prevent backflow of the drug upon removal. Remove the internal cannula and replace the dummy cannula.

## Method validation

**Fig. 3:**
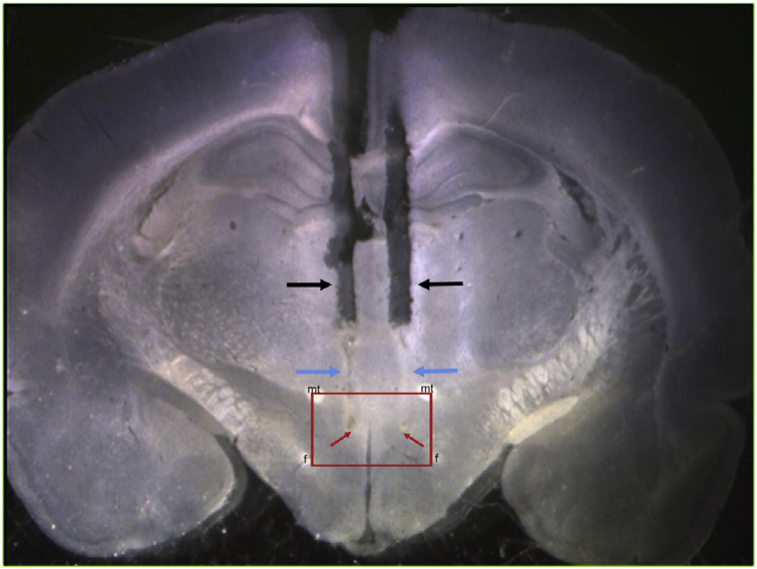
Light microscope image of a coronal brain slice illustrating the guide and internal cannula tracks (black and blue arrows, respectively). The cannulas are considered to be in the DMH if the tips (indicated by the red arrows) are located ventral to the mammilothalamic tract (mt), medial to the fornix (f), and between 2.6 to 3.8 mm posterior to bregma (this image is from (1) and illustrates cannula tracks from an animal used in that study).

**Fig. 4:**
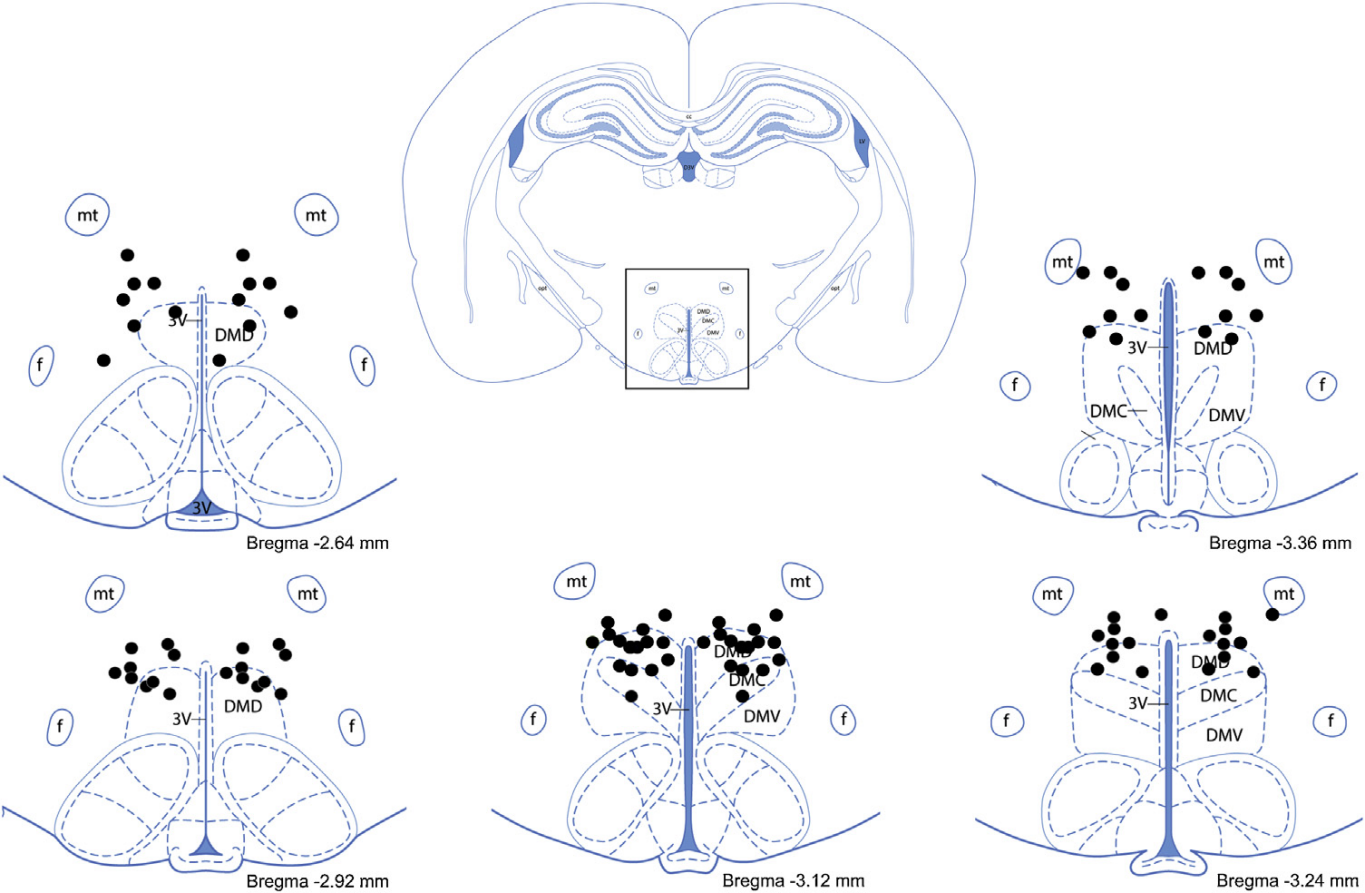
Rat brain atlas images (adapted from [3]) illustrating successful injection sites. Sample rat brain image of a coronal section including the DMH (top, center) with the box indicating the general region of the DMH. Successful injection sites are plotted at −2.64 mm, −2.92 mm, −3.12 mm, −3.24 mm, and −3.36 mm posterior to bregma (bottom; data points taken from (1)). Labelled structures are as follows: dorsal region of the DMH (DMD), compact region of the DMH (DMC), ventral region of the DMH (DMV), third ventricle (3 V), mammillothalamic tract (mt), fornix (f), opt (optic tract).

This procedure yields an 81% success rate. To verify whether cannulas are successfully implanted for drug injection into the DMH, fix brains via transcardial perfusion, and store them in 4% paraformaldehyde in phosphate buffered saline (PBS) for 24 h, followed by incubation in 30% sucrose in PBS for 24 h, both at 4 °C. Remove brains from the sucrose solution and place them in a rat brain matrix designed to hold brains from rats weighing between 35–175 g. Beginning 1 mm anterior to the optic chiasm, cut a 6 mm section containing the hypothalamus using razor blades and mount it with the posterior side facing down on a freezing block at −50 °C. Coat the brain with Clear Frozen Section Compound and wait for it to freeze. Using a cryostat, cut 30 μm slices and place them in 24-well plates containing 0.02% sodium azide in PBS and store them at 4 °C. To determine whether cannulas are successfully implanted in the DMH, view brain slices showing clear cannula tracks in a petri dish containing PBS under a stereo microscope (Fig. 3). Using the adult rat brain atlas as a reference [8], plot cannula tips from clear, representative slices to determine their exact location. Consistent with reports in adult animals, cannulas can be considered to be localized in the DMH if the tips are medial to the fornix, ventral to the mammilothalamic tract and between 2.6 and 3.8 mm posterior to bregma (Fig. 4) [4,9]. If a cannula is found to be outside of this area, the behavioural data from that animal should be excluded from statistical analyses.
